# A peek behind the curtain in the diagnosis and management of COVID‑19‑Associated Mucormycosis (CAM)

**DOI:** 10.1186/s42506-022-00125-1

**Published:** 2023-03-02

**Authors:** Nermin A. Osman, Mohammed Moustapha Anwar, Bivek Singh, Girish K. Gupta, Amgad M. Rabie

**Affiliations:** 1 Biomedical Informatics and Medical Statistics Department, Medical Research Institute, Alexandria University, Alexandria, Egypt; 2 Department of Biotechnology, Institute of Graduate Studies and Research (IGSR), Alexandria University, Alexandria, Egypt; 3 Tongji University, Shanghai, China; 4 Department of Pharmaceutical Chemistry, Sri Sai College of Pharmacy, Badhani, Pathankot, 145001 Punjab India; 5 Drug Discovery & Clinical Research Department, Dikernis General Hospital (DGH), Magliss El-Madina Street, Dikernis City, 35744 Dikernis, Dakahlia Governorate Egypt

**Keywords:** Mucormycosis, COVID-19, CAM, Multidisciplinary team, Antifungal therapies, Antimicrobial resistance, Complete surgical debridement, Antifungal stewardship

## Abstract

Coronavirus disease 2019 (COVID-19)-associated mucormycosis (CAM) is responsible for a high mortality rate due to its unique and severe host-pathogen interactions. Critically ill or immunocompromised COVID-19 patients are more prone to suffer from aggressive mycoses. Probable victims include those with uncontrolled diabetes mellitus (DM), metabolic acidosis, prolonged neutropenia, increased ferritin levels, hypoxia, and prolonged hospitalization with/without mechanical ventilators and corticosteroids administration. The current review aims to outline the journey of patients with CAM as well as the advantages and disadvantages of the currently available diagnostic techniques. It also discussed the current status of treatment options and caveats in the management of mucormycosis. Multidisciplinary team, early diagnosis, controlling the predisposing condition(s), complete surgical debridement, effective antifungal therapies (e.g., amphotericin B, isavuconazole, and posaconazole), and implementing antifungal stewardship programs are imperative in CAM cases.

## Introduction

Mucormycosis leads to poor survival rates regardless of the notable understanding of its pathophysiology, enhanced diagnostic tools, and different treatment options [1, 2]. Mucormycosis or the ‘black fungus’ infection (‘black fungus’ is a metaphoric, not scientifically accurate, nomenclature) is a member of the order Mucorales and is a very rare but serious angioinvasive disease, with 11 genera and approximately 27 species that can cause human infections [3]. Mucormycosis is a noncontagious disease (human-to-human transmission does not occur in normal conditions); humans mainly acquire the infection through inhalation of the sporangiospores, and occasionally through traumatic inoculation or ingestion of contaminated food. *Rhizopus oryzae* (*R. oryzae*) fungus accounts for nearly 60% of mucormycosis in humans around the world, being responsible for 90% of the rhino-orbital-cerebral mucormycosis (ROCM) type [4]. *Lichtheimia, Apophysomyces, Cunninghamella, Rhizomucor,* and *Mucor* species are less common types [5]. Mucorales along with other moulds invade airways, disrupt mucosal and skin barriers and natural host defenses, and have common histopathological and clinical features [6, 7]. For instance, *R. oryzae*, *Lichtheimia, Rhizomucor,* and *Mortierella* spp. can infect patients with diabetic ketoacidosis (DKA) or other types of acidosis. With their distinctive host-pathogen interactions, Mucorales evade the host immune system and facilitate disease progression regardless of treatment, increasing the mortality rate [8].

According to the World Health Organization (WHO) statistics, mucormycosis occurs with a global incidence rate of 0.005-1.7 per million population with a case fatality rate of 46%. In India and China, the incidence of mucormycosis predominantly increases among patients with uncontrolled diabetes mellitus (DM) [9–12]. In India only, the prevalence of mucormycosis is roughly 80 times (about 0.14 cases/1000 population) higher than that in developed countries, because India ranks second with more than 77 million people with DM, which might be the most important factor (followed by the inadequate infection prevention and control measures in hospitals) for the high prevalence of cases there [12–14]. Critically ill or immunocompromised coronavirus disease 2019 (COVID-19) patients are more liable to suffer from aggressive mycoses [15]. Examples for coexisting conditions that aggravate the aggressiveness of mycoses are the uncontrolled DM, metabolic acidosis and DKA, prolonged neutropenia, increased ferritin levels, hypoxia, prolonged hospitalization with/without mechanical ventilators, trauma, use of corticosteroids, hemopoietic malignancy, immunosuppression associated with a reduced phagocytic activity of white blood cells (WBCs), solid organ transplantation, and allogeneic hematopoietic stem cell transplantation [16–18].

**Fig. 1:**
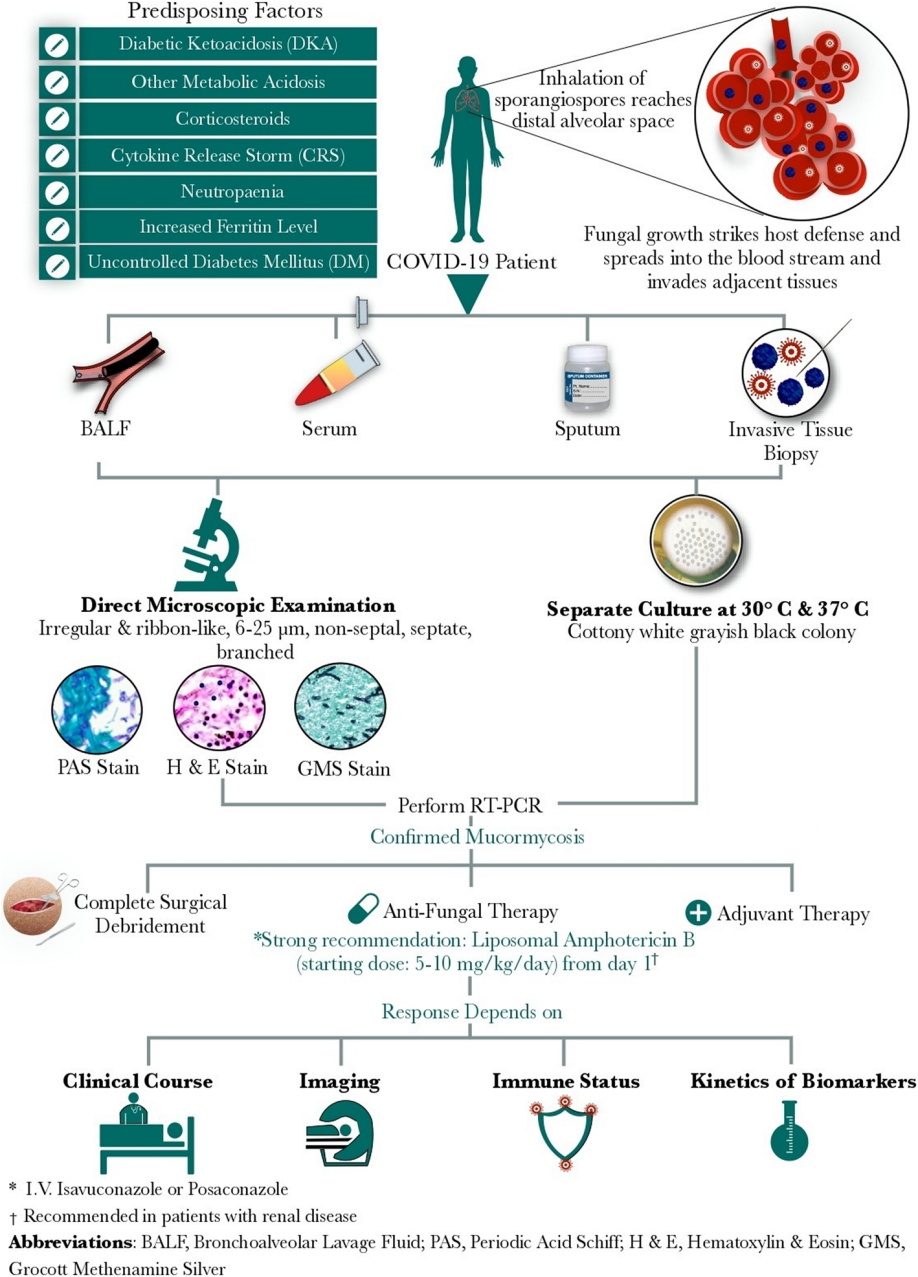
A schematic chart summarizing the complete pathogenic, diagnostic, and therapeutic journey of CAM patients

Recent global medical reports have documented that the rate of mucormycosis increases in COVID-19 patients and the cases are suffering from significantly poor prognoses [18]. In a recent systematic review, 93% of 41 COVID-19 patients with confirmed mucormycosis were diabetic, while 88% received corticosteroids [19]. Similarly, Singh *et al.* have confirmed the diagnosis of mucormycosis in 95% of COVID-19 patients, whereas 80% had DM, and about 76% received corticosteroids [18, 19]. Forty-six percent of confirmed mucormycosis cases already received corticosteroids within one month prior to diagnosis [13]. Prakash *et al.* also reported that 57% of patients had uncontrolled DM while 18% had DKA in the 2019 nationwide multicenter study of 388 suspected or confirmed cases of mucormycosis in India before COVID-19 [9]. Moreover, Ahmadikia *et al.* highlighted that COVID-19-associated mucormycosis (CAM) is more serious than influenza-associated mucormycosis, irrespective of an intense therapeutic approach [20]. Retrospective analysis of severe acute respiratory syndrome (SARS), global data, and other reports from China concerning influenza revealed that COVID-19-related fungal coinfections may be under/misdiagnosed [15]. The present review article highlights the main diagnostic criteria and the proper therapeutic options in COVID-19 patients who are confirmed with mucormycosis. Figure 1 outlines the COVID-19 patient's journey with mucormycosis, including patient's criteria, diagnostic tools, and management.

## Current Challenges in Diagnosis of Mucormycosis

The nonspecific and confusing clinical presentation of mucormycosis remains burdensome, making its diagnosis and treatment more difficult [21]. Mucormycosis can virtually affect any organ (e.g., central nervous system [CNS] in general, brain, nose, sinuses, jaw bones, skin, joints, heart, kidneys, lungs, gastrointestinal tract, and invasive mediastinum) [22, 23]. Just prior to the emergence of COVID-19 pandemic, the recent global guidelines for the diagnosis and management of mucormycosis in 2019 highlighted that diagnosis of mucormycosis is usually delayed with the rapid disease progression [16, 24]. This delayed diagnosis worsens in CAM cases due to many reasons, such as the difficulty in taking invasive tissue biopsies and the unease of aerosol-generating procedures in oral and maxillofacial surgery in COVID-19. Moreover, the laboratory diagnosis of mucormycosis is difficult since blood cultures are negative and their assessment is often feasible only after a relatively long period of time [25]. Unfortunately, although blood culture, histopathology, and direct examination are essential to diagnose mucormycosis, they are insensitive and time-consuming [26]. These low-sensitivity and slow procedures are certainly more apparent in COVID-19 cases.

Rapid diagnosis is usually deterred due to the relative lack of specific agents that detect mucormycosis in the cerebrospinal fluid [27]. In addition, early and rapid diagnosis of mucormycosis is relatively unusual from the historic point of view and according to the literature, since about half of the cases with mucormycosis were diagnosed only in the postmortem autopsy examination (i.e., after death) [28]. Namely, a 12-hour delay in the diagnosis of mucormycosis could be deadly. Hence, a timely diagnosis upon suspicion of mucormycosis, a proper referral to a top-level health care facility, and initiation of prompt antifungal treatment (specially, antimucormycosis therapies) would prevent tissue invasion and its damaging sequelae in COVID-19 patients [27]. Subsequently, this would minimize the impact of corrective surgery and improve survival and outcome [10, 29].

### The Clinical Criteria of Mucormycosis in COVID-19 Patients

Patients with COVID-19 exhibit ROCM as the most common clinical presentation seen worldwide in clinical microbiology [20, 23]. Thrombosis, eosinophilic necrosis of the underlying tissue, and giant cell invasion are hallmarks of mucormycosis, but diagnosis based on inconsistent symptoms/signs and clinical presentation is insensitive and nonspecific [15, 18, 30, 31]. Smith and Krichner established the gold standard criteria for the clinical diagnosis of mucormycosis in a report of three cases in 1958 (Table 1) [21]. Song *et al.* suggested to assess the risk factors, clinical settings, forms of invasive mycosis, advantages and limitations of diagnostic techniques, and demand for individualized or standard treatment options in patients with COVID-19 [15].

**Table 1:** The pathognomonic criteria for the clinical diagnosis of mucormycosis established by Smith and Krichner (Smith-Krichner's mucormycosis pathognomonic criteria) [21]

A short duration blood-tinged nasal discharge (usually dark-colored) on the side of facial pain	
A soft perinasal/periorbital swelling that progresses to discolouration and induration—with progressive vascular occlusion	
Blepharoptosis and globe proptosis, dilation and fixation of the pupil, and functional limitation	
Progressive lethargy, despite better response to antidiabetic medications	
Black necrotic turbinate, easily confused with dried blood	
Loss of corneal reflex and onset of facial weakness—often observed late during invasion	

Depending on the involved organ, ROCM ranges from a limited invasion to sinonasal tissue and rhino-orbital disease to a diffuse rhino-orbital-cerebral (ROC) disease that involves CNS [31]. For instance, patients with uncontrolled DM and DKA frequently present with ROCM, whereas those with neutropenia, organ and bone marrow transplant, and hematological malignancies usually exhibit pulmonary involvement. Diagnosis of mucormycosis relies on different integrative factors, such as the availability of imaging techniques (e.g., magnetic resonance imaging and computed tomography), comprehensive mycological and histological assessments, and qualified personnel [16]. Several studies suggested that real-time polymerase chain reaction (RT-PCR), radiological imaging, and culture used for invasive aspergillosis (IA) are also applicable if mucormycosis is suspected [32–37].

### Diagnostic Techniques in Mucormycosis: Strengths and Limitations

The United States Food and Drug Administration (FDA) has not yet approved serological assays that identify Mucorales compared to Aspergillus galactomannan index and β1,3-D-glucan (BDG) assays that can diagnose IA and other hyalohyphomycetes [35, 38]. Direct microscopy and/or fluorescent brighteners from clinical specimens (e.g., skin lesions, sputum, and bronchoalveolar lavage fluid [BALF]) usually suspect mucormycosis [26]. Microbiological identification (e.g., irregular and ribbon-like, 6–25 μm, nonseptate/pauci-septate, and branching pattern) of the Mucor hyphae distinguishes it from other fungi (i.e., invasive cryptococcosis, aspergillosis, and candidiasis) [15, 18, 31, 39]. Importantly, the ribbon-like and broader identity of the Mucorales hyphae are more authentic than its branching angle and septations [39]. However, data remain scarce to confirm the accuracy of using these criteria to distinguish Mucorales from other moulds. Furthermore, molecular/*in-situ* identification methods or culture of specimens are highly advocated to diagnose tissue mucormycosis [16].

Stains of fixed sections showing invasive nonpigmented hyphae, such as periodic acid-Schiff, hematoxylin-eosin, and Grocott-Gomori’s methenamine-silver stain, are confirmatory of mucormycosis [39]. Moreover, separate culture of specimens at 30 °C and 37 °C is highly recommended to identify genus and species, showing cottony white or greyish-black colony. Then, the morphological identification of fungi or DNA sequencing could be performed [35]. Nevertheless, Gomez *et al.* suggested that PCR should be restricted to tissues showing Mucorales hyphae upon staining to avoid the false positive results [40].

#### Lateral Flow Immunoassay (LFIA)

Lateral flow immunoassay (LFIA) is an essential serological assay for large-scale screening of immune responses to severe acute respiratory syndrome coronavirus 2 (SARS-CoV-2), being used for national and regional seroprevalence surveys in Europe and the USA [41, 42]. LFIA is a rapid, specific, accurate, cheap, and an easy-to-use test that early detects cell wall fucomannan of Mucorales in clinical samples, such as serum, urine, BALF, and tissue [43]. Furthermore, LFIA can be either performed by qualified health care professionals or self-administered. Despite some reports of high sensitivity and specificity, use of LFIA is relatively limited to date due to its variable degree of sensitivity (the high degree of sensitivity is broadly changeable and is not always constant to a certain reliable value among all cases) [44–49].

#### PCR of Fresh Tissues

The PCR of fresh tissues is highly sensitive and specific with PCR-restriction fragment length polymorphism that confirms the diagnosis to the genus or species level. The European Confederation of Medical Mycology (ECMM) states that the fresh tissue is more advantageous than those embedded in paraffin because formalin damages DNA [16]. However, lack of matching controls and DNA extraction and contamination of the specimen might lead to false negative results, whereas false positive results may arise due to fungal contamination of the PCR master mixture [4, 50–52]. Besides, obtaining a tissue biopsy might not be always feasible in vulnerable patients [35].

#### RT-PCR of Blood/Serum

The PCR of blood/serum samples can early identify circulating fungal DNA, help follow up and assess treatment response, and indicate angioinvasive disease. However, low quantities of fungal DNA, heterogeneity of internal transcribed spacer regions, and lack of both comprehensive database and species-specific probes provide false negative result. False positive results occurring due to amplification of DNA contaminating the sample are less likely compared to other samples [53–59].

## The Treatment of Mucormycosis: What We Already Know

Although investigators have evaluated numerous therapeutic regimens, mainly systemic glucocorticoids are proven to improve survival in hypoxemic COVID-19 patients [60]. Recognizing disease patterns upon early detection, rapid control or the possible discontinuation of predisposing factors (e.g., hyperglycemia), the fast surgical debridement of infected tissues, the early administration of the optimal dose of active antifungal agents (i.e., liposomal amphotericin B), and using adjuvant therapies are mainstay in the effective management of mucormycosis [16, 37, 61]. Management of mucormycosis during COVID-19 has encountered several hurdles, including multiorgan failure, the difficulty of controlling the hyperglycemia, lack of manpower in operating rooms, and the shortage in departmental resources. Moreover, angioinvasion with hematogenous dissemination, vessel thrombosis, and necrotic tissues impede the needed penetrability of immune cells and antifungal drugs [8, 16, 62–64]. Evaluation of antifungal response relies on clinical course, imaging, the patient's immune status, and kinetics of biomarkers (e.g., cytokines) [16, 65]. It is worth mentioning that severe COVID-19 patients often develop acute respiratory distress syndrome (ARDS), with significant elevations of C-reactive protein (CRP) and diverse inflammatory/immune cytokines like interleukins (ILs) 5, 6, 8, 10, 13, 17, and 22, as a result of hyperactivation of the innate and adaptive immune responses (cytokine release storm) [66]. Thus, regaining immune system balance and repressing markedly elevated inflammatory cytokines are very necessary for effectively treating severe COVID-19 patients and the accompanying mucormycosis infection.

Upon suspicion of mucormycosis, the ECMM and Mycoses Study Group Education and Research Consortium (MSG-ERC) in 2019 strongly recommended an appropriate imaging, followed by an immediate complete surgical intervention whenever possible, and systemic antifungal drugs. Surgical debridement for both necrotic tissue and neighboring healthy-looking infected tissues should be aggressive when needed. Surgery is helpful in soft tissue and ROC infections, and it may be valuable in a single localized pulmonary lesion. Table 2 outlines the latest ECMM/MSG-ERC treatment recommendations. Data are still somewhat insufficient to support the use of antifungal combination therapy (i.e., polyenes/azoles or polyenes/echinocandins) [16, 48, 67, 68]. Treating physicians should quickly taper CAM predisposing/aggravating drugs (e.g., corticosteroids and immunosuppressive drugs) to the lowest possible dose. Combined with antifungal therapy, hyperbaric oxygen as an adjunctive therapy provides an oxygen-rich cellular environment with cytokines. For instance, interferon-*γ* and/or granulocyte-macrophage colony-stimulating factor might improve the immune system against certain Mucorales *in vitro* [69, 70].

**Table 2:** The updated ECMM/MSG-ERC treatment guidelines for mucormycosis [16]

Drug(s)	Position of Treatment	Strength of Recommendations
Amphotericin B lipid complex, high-dose liposomal amphotericin B^*^	First-line monotherapy	Strong
Isavuconazole: intravenous or oral^†^	First-line or Salvage	Moderate
Posaconazole: intravenous or delayed-release tablets^†‡^	Salvage	Moderate
Posaconazole: oral suspension^**^	First-line monotherapy	Marginal^†^

**N.B.** Amphotericin B deoxycholate is discouraged, because of significant toxicity—but it may be the only option in limited-resource settings. In cases of failure with isavuconazole or posaconazole, all three lipid-based amphotericin B formulations are recommended (i.e., moderate-to-strong). * Five to ten mg/kg/day of liposomal amphotericin B as a first-line treatment is strongly advocated irrespective of involved organs. The dose can be reduced if significant renal toxicity develops, but those less than five mg/kg/day are recommended but with marginal strength. ** Particularly when formulations with higher exposure are readily available. † Posaconazole or isavuconazole may be used as maintenance therapy. ‡ Primary prophylaxis with posaconazole may be recommended in neutropenic patients, those with graft-versus-host disease (GVHD), or high-risk factor

Among other antifungal therapies, amphotericin B possesses the highest activity except for some *Cunninghamella* and *Apophysomyces* isolates. A minimum inhibitory concentration (MIC) of ≤0.5 *μ*g/mL amphotericin B significantly resulted in better outcomes over six weeks in humans [71]. Moreover, in patients with probable or confirmed mucormycosis, a combination of surgery with liposomal amphotericin B (10 mg/kg/day) for the first month of treatment, resulted in overall response rates of 36% and 45% at week 4 and week 12, respectively [67]. Similarly, isavuconazole and posaconazole are active, while some strains show a certain degree of susceptibility to terbinafine and itraconazole [72–76]. For instance, posaconazole had higher efficacy against strains of *R. oryzae* in infected mice [77]. Oral formulations of isavuconazole and posaconazole are favored because they can be administered for a long period (e.g., several months), if required. Based on the findings of some recent studies that proved the roles and effects of prior excessive zinc intake (even from multivitamins taken during COVID-19 treatment) and higher blood glucose level in the severity of CAM cases [18, 78], a new additive therapeutic hypothesis for CAM can be established in the current work. This hypothesis states that zinc ion-sequestering agents and blood glucose-lowering agents (e.g., most antidiabetic medicines) may have indirect positive roles in managing and accelerating the cure and recovery, along with reducing the mortality rates, of CAM. In Table 3, we have enumerated some of the obstacles that might face management of mucormycosis in COVID-19 patients.

**Table 3:** Caveats in the management of mucormycosis (including CAM)

Caveat	Reference(s)
Delaying treatment with amphotericin B in patients with hematological malignancies for >5 days causes about 2-fold increase in 12-week mortality.	[10]
The duration of active antifungal treatments has not been established yet, but weeks to months are generally advised.	[26]
Delaying surgery and the presence of multiorgan failure impede imaging procedures for COVID-19 patients with mucormycosis.	[62]
It is impossible to perform surgery in disseminated mucormycosis or when infection reaches lung parenchyma next to large vessels or some parts of the brain.	[26]
*Mucoraceous* fungi exhibit *in-vitro* resistance to most antifungals, including voriconazole.	[73–75, 79]
First-line treatment failure arises due to drug intolerance or refractory mucormycosis.	[16]
Assessment of treatment response may be difficult due to postoperative changes and scarring.	[16]
Isavuconazole can shorten the corrected QT (QTc) interval although it is less hepatotoxic.	[80–82]
Hyperbaric oxygen should be used cautiously due to the unavailable supportive clinical data.	[69, 70]

## Conclusion and Take-home Messages

Multidisciplinary team preparedness, early diagnosis, controlling the predisposing condition(s), applying complete surgical debridement, using systemic and local antifungal therapies, and implementing antifungal stewardship programs are imperative for CAM patients. Further research is mandatory to explore other noxious prognostic factors in COVID-19 associated mucormycosis and methods to reduce their influence on morbidity and mortality along with finding more effective medications.

## Data Availability

Not applicable.

## Abbreviations

BALF: Bronchoalveolar lavage fluid
BDG: β1,3-D-Glucan
CAM: COVID-19-associated mucormycosis
CNS: Central nervous system
COVID-19: Coronavirus disease 2019
DKA: Diabetic ketoacidosis
DM: Diabetes mellitus
ECMM: European Confederation of Medical Mycology
GVHD: Graft-versus-host disease
IA: Invasive aspergillosis
LFIA: Lateral flow immunoassay
MIC: Minimum inhibitory concentration
MSG-ERC: Mycoses Study Group Education and Research Consortium
QTc: Corrected QT
ROC: Rhino-orbital-cerebral
ROCM: Rhino-orbital-cerebral mucormycosis
RT-PCR: Real-time polymerase chain reaction
SARS: Severe acute respiratory syndrome
SARS-CoV-2: Severe acute respiratory syndrome coronavirus 2
WBCs: White blood cells
